# Oxytocin: Old Hormone, New Drug

**DOI:** 10.3390/ph2030168

**Published:** 2009-12-09

**Authors:** Jolanta Gutkowska, Marek Jankowski

**Affiliations:** Laboratory of Cardiovascular Biochemistry, Centre de recherche, Centre hospitalier de l’Université de Montréal (CRCHUM)–Hôtel-Dieum, Department of Medicine, Université de Montréal, Montreal, Quebec, Canada

**Keywords:** oxytocin, stem cells, heart, cardiomyogenesis

## Abstract

Oxytocin (OT), traditionally associated with reproductive functions, was revisited recently, and several new functions in cardiovascular regulation were discovered. These functions include stimulation of the cardioprotective mediators nitric oxide (NO) and atrial natriuretic peptide. OT’s cardiovascular outcomes comprise: (i) natriuresis, (ii) blood pressure reduction, (iii) negative inotropic and chronotropic effects, (iv) parasympathetic neuromodulation, (v) NO pathway involvement in vasodilatation and endothelial cell growth, (vi) anti-inflammatory and (vii) antioxidant activities as well as (viii) metabolic effects. In addition, we have reported abundant OT in the early developing heart with its capacity to generate cardiomyocytes (CMs) from mouse embryonic stem cells and stem cells residing in the heart. OT increases glucose uptake by cultured CMs, in normal, hypoxic and even in insulin resistance conditions. In experimentally-induced myocardial infarction in rats, continuous *in vivo* OT delivery improves the cardiac healing process and cardiac work, diminishes inflammation, and stimulates angiogenesis. Therefore, in pathological situations, OT plays an anti-inflammatory and cardioprotective role, enhancing vascular and metabolic functions, with potential therapeutic application(s).

## 1. Introduction

As early as 1910, Henry Dale wrote in *Biochemistry Journal* [1]: “It does not seem justifiable to draw…the conclusion that the principle (in pituitary body extracts) acting on the plain muscle of the uterus is different from that which acts on the arteries”. 

Ott and Scott [2] demonstrated that besides their effect on uterine activity, posterior pituitary extracts also promote milk ejection – the two principal activities of oxytocin (OT), the structure and synthesis of which were not elucidated until 50 years later by Du Vigneaud and co-workers [3]. OT, the most abundant hormone in the human body, is mainly produced in the paraventricular nucleus and supraoptic nucleus of the hypothalamus, and released from hypothalamic nerve terminals of the posterior pituitary into the circulation. It differs, by only two amino acids, from vasopressin (AVP), which is also produced in these nuclei and stored in the posterior pituitary. OT in the circulation was originally believed to stimulate uterine contractions to start parturition and milk-ejection during lactation. However, similar numbers of oxytocinergic neurons have been found in the male and female hypothalamus, and the same stimuli induce OT release in both genders, suggesting other physiological functions. In fact, OT receptors (OTR), widely expressed in several organs, elicit a variety of physiological responses [4], such as complex sexual and maternal behavior. Indeed, OT is also involved in cognition, tolerance and cardiovascular regulation. Our interest in the cardiac OT system emerged from longitudinal investigations into the role of the brain in the control of cardio-renal homeostasis [5]. These experiments led to the observations that OT and its OTR are synthesized in the human and rat heart [6,7] and that OT exerts cardioprotection either directly or *via* stimulation of mediators such as the natriuretic peptides (NPs) [6] and nitric oxide (NO) [8]. In addition, OT has been identified as a potent, naturally-occurring cardiomyogen, which, by upregulation of its own receptors in mouse embryonic stem (ES) cells [9,10] and stem cells isolated from the adult mouse and rat heart [11,12] promotes differentiation into functional cardiomyocytes (CMs). A recent study has disclosed that OT stimulates glucose uptake in rat CMs [13]. Consequently, OT emerges as a pleiotropic hormone involved in cardiovascular and metabolic functions.

## 2. Cardiac OT Actions

Although the pathophysiological role of OT is beginning to be understood, accumulating evidence indicates multiple beneficial effects in the heart and vasculature. To date, OT’s cardiovascular properties include: i. the induction of stem cell differentiation into CMs [9,10]; ii. natriuresis [14], and decreased blood pressure (BP), possibly secondary to atrial natriuretic peptide (ANP) release [6]. iii. negative inotropic and chronotropic effects [15] and parasympathetic neuromodulation [16]; iv. vasodilatation *via* the OTR-induced NO pathway; v. endothelial cell growth and possible vessel generation [17]; and vi. modulation of insulin release [18] and anti-diabetic actions. 

**Figure 1:**
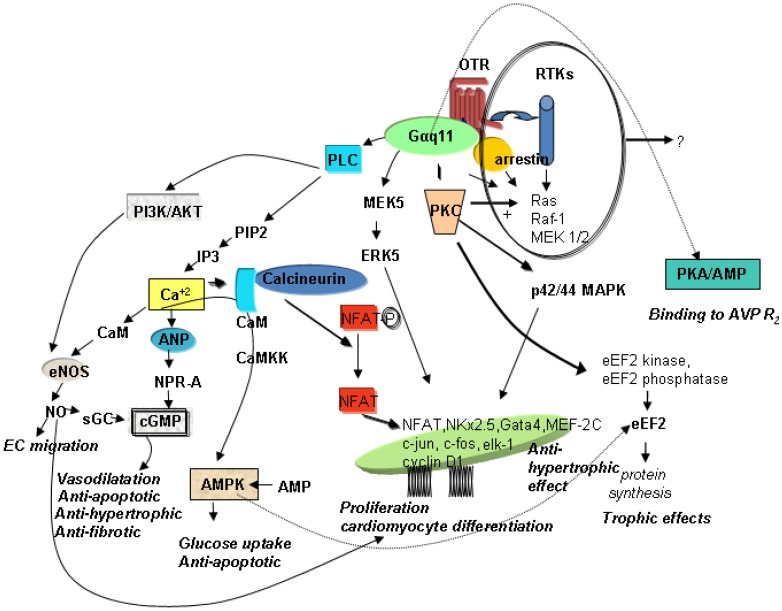
Schematic diagram of potential signalling pathways of OTR in CMs. AMPK—AMP—activated protein kinase; ANP—atrial natriuretic peptide; AVPR2—vasopressin receptor R2; CaM—calmodulin; CaMKK—Ca^+2^ calmodulin-dependent protein kinase; cGMP—cyclic guanosine monophosphate; CMs—cardiomyocytes; EC—endothelial cells; eEF2—eukaryotic translation elongation factor 2; eNOS—endothelial nitric oxide synthase; ERK—extracellular signal-regulated kinase; IP3—inositol triphosphate; MAPK−mitogen-activated protein kinases; MEK—MAPK/ERK; NFAT—nuclear factor of activated T-cells; NO—nitric oxide; NPR-A—natriuretic peptide receptor A; OTR—oxytocin receptor; PIP2—phosphatidylinositol 4,5-bisphosphate; PI3K—phosphatidyl-3 kinase; PKC—protein kinase C; PLC—phospholipase C; RTKs—receptor tyrosine kinases; sGC—soluble guanylyl cyclase.

OT’s effects are mediated by OTR, G protein-coupled receptors that contain seven transmembrane domains. In uterine cells, OTR transduce signalling primarily *via* Galphaq subunits to activate phospholipase C-beta and mitogen-activated protein kinase (MAPK). In cardiac cells, several signalling pathways have also been postulated in conjunction with specific functions in the heart. Figure 1 illustrates the hypothetical pathways in the heart that are associated with cardioprotection, such as the prevention of apoptosis, CMs hypertrophy, and fibrosis, with stimulation of glucose uptake, cell proliferation and differentiation.

**Figure 2:**
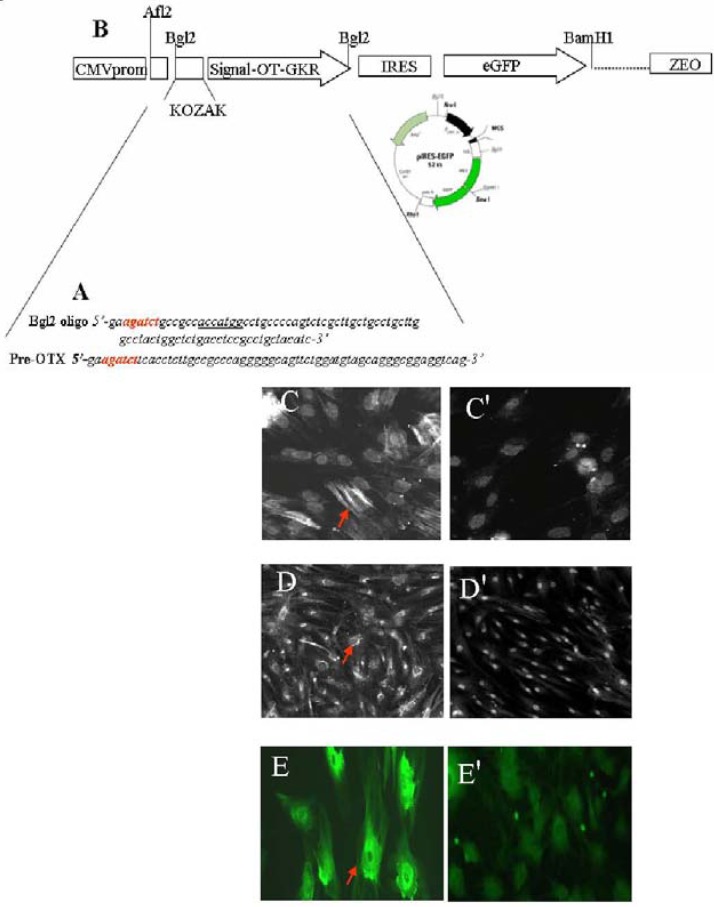
OT treatment of stem cells stimulates vascular cell markers. The sequence of oligonucleotides presented in (**A**) were used for: Construction of sites sensitive for restriction enzyme Bgl2 used for cloning (red); construction of consensus sequence for initiation of translation coding sequences (KOZAK, underlined) and, the generation of region coding OT-Gly-Lys-Arg (OT-GKR) oligopeptide. Oligonucleotide ligation, PCR amplification and molecular cloning in bacterial vector were performed in collaboration with Dr. Dominic Devost and Dr. Hans H. Zingg, McGill University. (**B**) The construct was introduced into internal ribosome entry site (IRES) vector containing the cytomegalovirus (CMV) promoter, the green fluorescence protein (eGFP) sequence and zeomycin resistance gene. This construct was employed for transfection of human mesenchymal cells. For transfection, 2 µg plasmid DNA was mixed with FuGENE 6 Transfection Reagent (Roche Diagnostics, Indianapolis, IN, USA: Cat. No. 11 815 091 001) according to the manufacturer’s specifications. Cells expressing OT-GKR protein produced smooth muscle actin (**C**) and vascular endothelial growth factor (**D**), as detected by immunofluorescence. Control mesenchymal cells transfected with the vector were negative (**C'** and **D'**, respectively). Vascular endothelial growth factor production was also stimulated by OT treatment of stem cell fractions isolated from the rat heart as side population (E), compared to non-treated controls (**E'**).

In addition, this signalling depends on coupling to specific G-proteins, cell type, and localization on the cell membrane surface. As a result, OTR stimulate different second messengers which, consequently, exert various physiological effects [19]. Due to its organ- and tissue-specific expression patterns, it is believed that OTR is regulated largely at the gene transcription level [20]. In the cardiovascular system, OTR are associated with the ANP-cGMP and NO-cGMP pathways, which reduce the force and rate of contraction and increase vasodilatation. In addition, OT and the other neurophyseal hormone AVP can evoke similar effects in some organs [4], including the differentiation of stem cells into CMs [21,22]. The absence of either OT or its receptors in knockout mice, however, has not been reported to produce cardiac insufficiencies [23]. Although OT knockout mice have a normal heart structure, experiments have shown augmented intrinsic heart rates in these animals, indicating that an intracardiac OT system controls cardiac electrical activity [24,25]. Correspondingly, our studies have demonstrated that OT slows heart rate and contractility *via* stimulation of the cardiac cholinergic system and NO [15,16]. These OT outcomes can be recognized as being beneficial to the heart. At the cellular level, protective OT has: (i) antioxidant properties [26] and (ii) anti-inflammatory actions [27], (iii) potentiates glucose uptake in neonatal and adult CMs exposed to hypoxia and conditions of insulin resistance mimicked by the presence of ketone bodies [13], (iv) stimulates endothelial markers in mesenchymal cells and stem cells isolated from the heart as a side population (Figure 2). 

The different cardioprotective actions of OT were recently demonstrated in animal models of myocardial infarction (MI). In rat, rabbit and pig models of ischemic heart disease, OT treatment significantly reduced infarct size and improved parameters of heart function [28,29,30,31].

## 3. Mechanisms of OT-Mediated Cardioprotection

OT’s negative chronotropic action was recently associated with attenuation of cardiac damage evinced by ischemia-reperfusion [30]. Therefore, OT, by activating intrinsic cardiac cholinergic neurons and NO release [16], can effectively inhibit cardiac sympathetic nerve activity and improve left ventricular ejection fraction in rats subjected to MI. Positive cardiac effects can also be attributed to the fact that OT stimulates ANP release from isolated, perfused hearts [6] by improving hydromineral homeostasis as well as cardiac hypertrophy and reducing pro-inflammatory mediators. ANP, a member of the NPs family that includes BNP, C type natriuretic peptide, and urodilatin, is released into the circulation after volume expansion, atrial stretch [32], hypoxia [33] and in response to various hormones and neurotransmitters [32]. ANP causes BP to decline with a concomitant increment of diuresis, natriuresis, and decrease of plasma volume [34]. NPs also inhibit the sympathetic nervous system and hormones involved in cardiac hypertrophy, such as angiotensin II, endothelin and AVP. NPs signalling *via* functional receptors (NPR-A and NPR-B) prevents pathological hypertrophy [35] and cardiac fibrosis [36] by attenuating both DNA and collagen synthesis in cardiac fibroblasts, oxidative stress [37] and inflammation [38]. Recent reports indicate that BNP and ANP activity is associated with lipolysis and postprandial lipid oxidation [39]. Both hormones modulate fatty acid trafficking and prevent triglyceride accumulation in CMs *via* cGMP signalling [40]. These physiological functions, altered in diabetes and obesity, may reflect the diminished plasma NPs levels seen in obese people [41,42]. Obesity is also associated with OTR knockout in mice, but it remains to be determined why this effect is observed only in male, but not female animals [23].

Our recent report shows that OT increases glucose uptake in CMs *via* phosphoinositide-3-kinase (PI3K) and potentiates the glucose uptake effect of 2,4-dinitrophenol, an uncoupler of oxidative phosphorylation targeting the mitochondria [13]. PI3K pathways are considered beneficial during myocardial injuries [43,44]. The calcium-calmodulin kinase kinase (Ca-CAMKK)-AMP-activated protein kinase (AMPK) pathways are also involved in OT-mediated glucose uptake in skeletal muscles [45] and CMs [13]. AMPK activation in the heart after ischemia and reperfusion is recognized as cardioprotective because AMPK limits apoptosis and cell damage [46,47]. In this regard, Kewalramani *et al.* [48] have defined AMPK as a powerful cardiac anti-inflammatory protector against tumor necrosis factor-alpha (TNFα)-triggerred CMs apoptosis through phosphorylation of Bad protein, subsequently suppressing mitochondrial apoptotic signalling. In addition, AMPK and CAMKK activation stimulates the phosphorylation of various downstream kinases, including eukaryotic elongation factor 2 kinase involved in the reduction of CMs enlargement [49]. AMPK can also suppress pathological cardiac hypertrophy [50]. On the other hand, in endothelial cells, the pro-angiogenic effect of OT required activation of the PI3K/AKT (protein kinase B)/eNOS (endothelial nitric oxide synthase) pathway [51]. We should also consider p38 MAPK and extracellular signal-regulated kinase 1/2 (ERK 1/2) phosphorylation which may contribute to OT’s proliferative activity [52]. More recently, in a rabbit model of myocardial ischemia-reperfusion, OT treatment induced ERK1/2, AKT and eNOS phosphorylation in cardiac tissues [29]. Therefore, OT, like other G-protein-coupled ligands, can act by PI3K/AKT activation and projection onto downstream kinases, such as glycogen synthase kinase-3 beta, targeting the mitochondria to protect cells [53]. 

## 4. OT’s Role in Inflammation

The anti-inflammatory properties of OT have been observed in the early stages of research on OT [54], and its role in the modulation of immune and inflammatory responses is supported by the fact that the entire OT system, OT and functional OTR, are expressed in the thymus network [55,56]. Furthermore, the OTR gene contains acute phase reactant and interleukin response elements [57]. Several works have documented OT’s anti-inflammatory action in humans and animal models. Petersson *et al*. [58] noted that OT decreased carrageenan-induced edema and neutrophil recruitment. The anti-inflammatory effects of OT have been demonstrated in experimentally-induced ulcer and colitis in rat and guinea pig models [59]. OT protects against sepsis-evoked multiple organ damage, supporting the potency of its anti-oxidative action in injured tissues [60] Furthermore, OT has been shown to improve the anti-oxidative stress of colonic tissue and ameliorate oxidative colonic injury *via* a neutrophil-dependent mechanism [26]. OT also improves skin damage and oxidant gastric injury in rats exposed to high temperature insult. Subcutaneous OT administration reverses burn-induced increases in malonedialdehyde and myeloperoxydase as an index of tissue neutrophil infiltration, and reduces gastric damage [61]. OT treatment before or immediately after hepatic ischemia-reperfusion significantly reverses transaminase and TNFα elevation in the circulation [62]. OT administration to hamsters blocks stress-induced increases in cortisol levels and facilitates wound-healing [63]. Interestingly, social interactions of animals during OT treatment promote additional benefit most likely by the release of other factors that may facilitate the effect of endogenous OT [64]. Several subsets of T cells, such as CD4^+^ and CD8^+^, express OTR mRNA, indicating an important role of the OT system in the response of these immune cells [56]. T cell infiltration is accompanied by monocyte/macrophage infiltration [65]. Therefore, by primarily affecting T cells, OT can also limit monocyte/macrophageinfiltration, as reported in our recent study [66]. On the other hand, the presence of OTR in monocytes and macrophages suggests that these cells are direct targets of OT in inflammation [27,56]. OT binding to OTR on pre-T cells elicits rapid phosphorylation of focal adhesion-related kinases [67]. This could also play a major role in the promotion of "immunological synapses" between immature T lymphocytes and antigen-presenting cells (e.g., macrophages). MI is associated with the elevation of pro-inflammatory mediators and acute deregulation of the immune system [68]. In the context of MI, a deregulated immune system is recognized as a decisive factor amplifying an excessive and unnecessary inflammatory response. Recent experiments indicate an inhibitory effect of OT on interleukin-6 (IL-6) expression in infarcted sites of rat hearts. IL-6 synthesis is rapidly induced in mononuclear cells and CMs of the ischemic myocardium [69]. This finding is consistent with studies demonstrating inhibition of IL-6 release by OT in pituitary cells [70] and most recently in macrophages and endothelial cells [27]. As in the uterus of pregnant rats, OT can activate T helper type 2 cells, stimulating IL-10 and suppressing T helper type 1 cells responsible for TNFα synthesis, regulation that is important for parturition [71]. Interestingly, regulation of appropriate cytokine genes can be executed *via* the cardioprotective calcineurin/nuclear factor of activated T-cells pathway triggered by elevated uterine OT [72].

## 5. OT Induces Stem Cell Differentiation

Hormonal treatment may, in its simplest form, induce mammalian stem cells into a special cell type that retains the ability to self-renew (*i.e.*, undergo cell division in an undifferentiated state) indefinitely and to differentiate into specialized cardiac cells. Thus, stem cell differentiation can protect the heart and combat pathologies related to CMs loss. Several observations brought us to the concept that the OT system could participate in activation of the stem cell pool residing in the heart and contribute to cardiac regeneration. 

Having encountered elevated OT levels in fetal and newborn hearts at a stage of intense CMs hyperplasia [10], we hypothesized a role for OT in CMs differentiation. After our initial study showing that OT induces differentiation of P19 embryonic carcinoma cells into cardiac muscle [9], numerous reports confirmed the effect in different lines of ES cells [8,73,74,75]. Some observations point to a mechanism involved in this process. Ca^+2^ mobilization in response to OT treatment has been detected in D3 ES cells differentiating into CMs [76]. It has also been shown that OT-induced differentiation of P19 stem cells into CMs is inhibited by the NOS inhibitor N(G)-nitro-L-arginine methyl ester (L-NAME). The NO donor *S*-nitroso-*N*-acetylpenicillamine (SNAP) was able to reverse L-NAME-mediated inhibition of P19 cell differentiation into CMs [8]. This study clearly indicates a role for NO and NOS enzymes in stem cell differentiation, but what is evident is that it may be a complex process. This complexity is highlighted by the fact that suppression of NOS activity by L-NAME has also been shown to increase the number of stem and progenitor cells differentiating into CMs [8]. Another study has reported that exposure of D3 stem cells to AVP augments the number of beating embryoid bodies and also heightens GATA-4 expression. These AVP effects on the cells were also found to be antagonized by L-NAME [21], again suggesting a positive role for NO in stem cell differentiation into CMs. This investigation highlighted the expression of AVP receptors in undifferentiated D3 cells, with the expression profile changing during the differentiation process [21]. It has been observed in the P19 cell model that AVP not only increases spontaneously-occurring cardiomyogenesis but also initiates the process [21,22].

The OTR-NO-cGMP pathway that is essential for OT-elicited differentiation of P19 stem cells into CMs is associated with elevation of GATA-4 and myocyte enhancer factor 2c (MEF2c) [8]. GATA-4 regulates the expression of genes that are critical for CMs differentiation. MEF2c is a member of the MEF2 family that is involved in cardiac, skeletal, and smooth muscle development. Partial GATA-4 gene targeting in cardiac and non-cardiac cells indicates that even modest variations in GATA-4 gene level or activity can play a role in the maintenance of normal cardiac function [77]. GATA-4 has also been implicated in intercellular cross-talk by inducing hypertrophy-associated angiogenesis *via* vascular endothelial growth factor (VEGF) release and targeting the endothelium [78]. GATA-4 also serves as a key transcriptional regulator of numerous cardiac peptides, including ANP, BNP and OTR [75]. GATA-4 has been also identified in stem and progenitor cells of the heart in combination with OT-mediated CMs differentiation [11,12]. A recent study has demonstrated that undifferentiated murine ES cells express BNP and its receptors, with its signaling being essential for cell survival and clonal growth [79]. This observation suggests possible interaction of the OT and NPs systems in ES cells during cardiomyogenesis. These results compelled us to hypothesize that OT treatment can be used for the renewal of CMs in damaged hearts.

## 6. OT Stimulates CMs Differentiation in 3-Dimensional Cultures

Multi-cellular complex aggregate formation and exposure to various agents promote the differentiation of P19 and embryonal cells or induce pluripotent stem cells to generate mesodermal or ectodermal lineages. Among the mesodermal derivatives formed in embryoid bodies, subtypes of cardiac cells (atrial CMs, ventricular CMs and pacemaker cells) have been identified by histological, molecular, and electrophysiological criteria [80,81]. 

**Figure 3:**
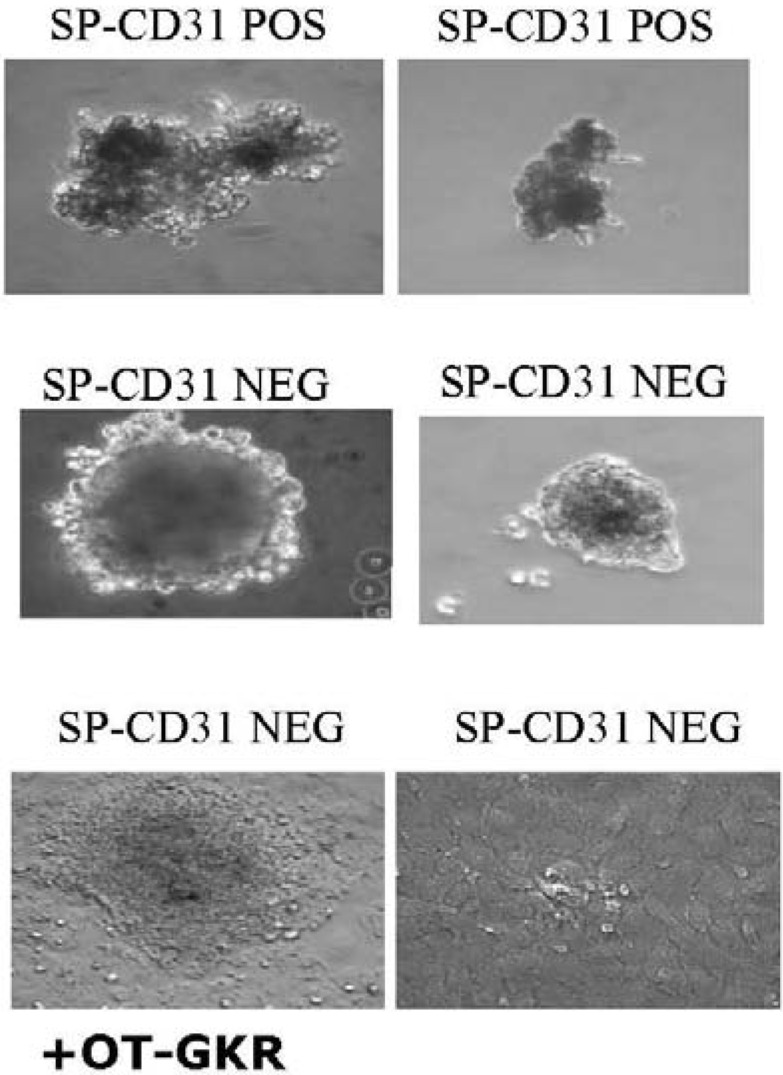
The presence of OT-Gly-Lys-Arg positively influences the shape and size of aggregates of the CD31 NEG subpopulation of side population cells (SPs) isolated from the rat heart.

P19Cl6 cells derived from P19EC cells seem not to be committed to a mesodermal lineage but rather represent a stage closer to differentiated cardiac muscle than the parental cell line. It was observed recently that OT does not induce cardiomyogenesis in monolayers of P19Cl6 cells, but does so in aggregates [75]. The presence of OT significantly influences the shape and size of aggregated stem cells isolated from the rat heart (Figure 3). This suggests that conditions inside aggregates, such as hypoxia, promote OTR and OT expression. Indeed, hypoxia efficiently influences the functionality of OTR in cardiac cells. Our data indicate that OT increases glucose uptake by CMs exposed to chemical hypoxia [13]. 

To determine whether the genetic modification of stem cells also stimulates cardiomyogenesis, the OT-Gly-Lys-Arg gene was inserted into D3 stem cells. In effect, we observed stimulation of spontaneously-beating embryoid bodies and predominant stimulation of cells expressing the ventricular electrophenotype and molecular CMs markers [76]. Interestingly, the elongated form of OT, OT-Gly-Lys-Arg, was the most potent cardiomyogen among all OT-like molecules investigated. These findings provide a new strategy for the regeneration of diseased hearts. Transgenic cells producing OT-Gly-Lys-Arg can be injected, for cardioprotection, in the myocardial infarcted rat model. Analysis of cardiac remodelling, scar reduction, hemodynamic and echographic parameters together with histochemical and molecular analyses will provide answers to whether or not these treatments can stimulate cardioprotection.

## 7. Differentiation of Endogenous Stem Cells Isolated from Animal Hearts

The idea that OT has cardioregenerative capacity is supported by the observation that this hormone induces the differentiation of cultured resident cardiac stem cells (CSCs) into CMs in mice [11] and rats [12]. In the adult heart, CSCs maintain a balance of survival, proliferation and self-renewal to replace mature cells that are lost during injury or turnover. Matsuura *et al*. [11] revealed the presence of a Sca-1^+^ stem cell population in adult mouse hearts expressing telomerase reverse transcriptase, which has been associated with self-renewal potential. These cells, lacking hematopoietic markers, are easily distinguished from hematopoietic stem cells of bone marrow origin, and when treated with OT, differentiate into CMs. Although the cells present the early cardiac markers GATA-4 and MEF2, they do not express Nkx-2 or genes encoding cardiac sarcomeric proteins. When exposed to OT, a small population of Sca-1^+^ cells manifest sarcomeric structures and form spontaneously-beating CMs. In addition, after intravenous delivery, Sca-1+ cardiac stem cells can home to the myocardium injured by ischemia/reperfusion and can functionally differentiate *in situ* [11]. Importantly, these cells had positive ionotropic responses to isoproterenol *via* β1-adrenergic receptor signalling. Given the apparently small number of CMs generated *in vitro* by OT stimulation, it raises the question whether or not OT-mediated cardiomyogenesis is a default pathway for CSCs. Accordingly, Matsuura *et al*. [11] reported that OT induces about 0.5-1% of Sca-1^POS^ ckit^NEG^ CD45^NEG^ cells from the adult murine heart to differentiate into functional, spontaneously-beating, immature CMs. On the other hand, a study by the same group in another CSC type isolated from the rat heart [12] disclosed that OT treatment resulted in the generation of 5% CMs. These cells, termed cardiac side population cells (CSPs), in contrast to corresponding side population cells (SPs) isolated from bone marrow, differentiated into CMs in response to OT treatment. Therefore, OT possesses more powerful cardiogenic activity against CSCs than previously reported. SPs have the ability to efflux Hoechst dye, a process dependent on ABC transporters. CSCs, especially Abcg2-dependent SPs, have been associated with stem/progenitor cells. These cells are positive for Abcg2, Sca-1, ckit (low), CD34 (low), CD45 (low) and negative for CD31 [82]. A possible role of CSCs in heart healing is indicated by increased numbers of Abcg2-expressing cells in the border zone adjacent to myocardial infarcts [83]. Stimulation of CMs differentiation could be concomitant with neovascularization because OT stimulates endothelial cell growth [17] and angiogenesis [51]. 

## 8. Summary and Conclusions

Our research has led to the observation that OT and OTR are synthesized in CMs, and we have identified OT as a potent, naturally-occurring cardiomyogenic factor, which, by OTR up-regulation, promotes the differentiation of embryonic and somatic stem cells residing in the heart to mature CMs. All these OT actions have physiological relevance, particularly on glucose uptake in CMs, since it is reduced in hearts from insulin-resistant diabetic mice, a disturbance that culminates in cardiac dysfunction.

Understanding the mechanisms of cardiac differentiation by OT can provide therapeutic approaches to the management of heart diseases. Currently, it is still extremely difficult to obtain new cardiac cells *in vitro* using stem cells isolated from the heart, as the only method that has provided satisfactory results is limited to co-culture with mature CMs. OT and some OT agonists, such as OT-Gly-Lys-Arg, that do not interfere with other physiological processes in the body (for example, without renal and hemodynamic effects), can successfully stimulate the differentiation of stem cell residing in the heart. In pathological conditions, such as cardiac ischemia and diabetes, this inducer can serve to stimulate the production of cardiac cells lost during these pathologies. The advantage of such a therapy is supported by the fact that OT is produced endogenously in the organism, and does not have significant side-effects when administered clinically. Moreover, it is now possible to inject (transplant) stem cells after previous stimulation with OT inducers, as in the case of heart attacks. Alternatively, direct treatment with OT molecules could promote cardiomyogenesis *in situ* and the regeneration of damaged hearts.
